# Structural and Physico-Chemical Changes of Mozzarella di Bufala Campana Cheese Influenced by Covering Liquid Composition

**DOI:** 10.3390/foods14091506

**Published:** 2025-04-25

**Authors:** Irene Fenga, Marcello Alinovi, Maria Paciulli, Germano Mucchetti, Emma Chiavaro

**Affiliations:** Food and Drug Department, University of Parma, Parco Area delle Scienze pad. 33, 43124 Parma, Italy; irene.fenga@unipr.it (I.F.); maria.paciulli@unipr.it (M.P.); germano.mucchetti@unipr.it (G.M.); emma.chiavaro@unipr.it (E.C.)

**Keywords:** Mozzarella cheese, buffalo milk, protein swelling, texture, rheology, water holding capacity, meltability, covering liquid, NaCl

## Abstract

Mozzarella di Bufala Campana is an Italian protected designation of origin cheese characterized by a stretched structure, high moisture (<65%), and short shelf life (<30 days). This cheese is generally stored refrigerated in a covering liquid that is an aqueous solution containing NaCl and organic acids. Although microbial growth has been reported as the main cause of quality deterioration, physico-chemical phenomena (water/solute migration, enzymatic reactions, etc.) also play a role in determining the cheese quality and its storability. This study investigates the effect of covering liquids formulated with different percentages of NaCl (1, 2%) and types of organic acids (lactic acid, citric acid, and a 1:1 mix of both) on the evolution of some physico-chemical characteristics of the cheese (moisture, pH, electrical conductivity, color, expressible serum, texture, rheology) during a 30-day storage period. Eight cheese batches collected from different dairies were considered as replicates of the study. The % of NaCl in the covering liquid showed a strong, significant effect on the evolution of different structural, physico-chemical characteristics of the cheeses; in particular, a NaCl concentration of 2% caused the greatest extent of moisture content increase because of casein swelling during storage, accompanied by softening of the structure.

## 1. Introduction

Mozzarella di Bufala Campana (MBC) PDO (Protected Designation of Origin) is a typical Italian cheese that is considered as the common ancestor of all the Mozzarella cheese varieties nowadays produced in the world. MBC can be both consumed as a fresh cheese, and used as an ingredient for other food preparations (e.g., pizza) [1]. At present, MBC cheese is produced from fresh buffalo milk that has to be processed within 60 h of the milking operations. Moisture content is usually higher than 60%, but limited to 65% by the PDO regulation. The typical MBC cheese structure is determined by the stretching of the cheese curd that has been acidified by the activity of autochthonous Lactic Acid Bacteria from the natural whey starter [2,3,4]. The resulting structure is characterized by overlying layers of casein fibers forming pockets that contain fat globules and serum channels. MBC cheese is also usually stored in a light brine, the covering liquid, usually composed of a slightly salty and acidic solution [5,6,7,8]. The above-mentioned characteristics are among those that determine the sensory profile of MBC, in terms of texture and juiciness of the cheese.

The expected shelf life of MBC cheese can vary from a few days to one month according to several technological parameters, with the most important ones being the entities of thermal treatments (i.e., milk pasteurization, curd stretching) and the cheese moisture content at the end of cheesemaking. As already stated, MBC cheese moisture content is a key point for the perception of the sensory properties, but also for the profitability of the cheesemaking process, since it strongly affects the actual cheese yield. Differently from high-moisture cow Mozzarella cheeses which usually lose a significant part of their moisture during the storage in covering liquid [9,10], MBC can absorb moisture from the covering liquid [11]. Therefore, MBC cheesemakers have to manage the curd stretching operation by balancing the initial cheese moisture and the shelf life, because of the PDO moisture limitation (<65%).

MBC shelf life, as for all the other Mozzarella cheese varieties, is also affected by microorganisms surviving the lethal effect of cheese curd stretching and/or post-contamination during the hardening step conducted by water dipping [12,13]. Residual enzymatic activities, deriving from both endogenous (i.e., plasmin) or exogenous enzymes (e.g., residual activity of rennet enzymes) can also affect the freshness of MBC [14]. The MBC PDO standard, differently from most of the cow’s milk Mozzarella cheese varieties, does not allow for the use of heat labile microbial clotting enzymes [15].

Although the use of covering liquid is useful to preserve some important characteristics of high-moisture Mozzarella cheeses, such as its delicate, soft structure and juiciness, it is also considered by MBC producers to be a limiting factor for MBC shelf life, as it may favor, for example, moisture absorption or mass exchange phenomena of solutes and microbial growth. Differently from cow Mozzarella cheese, for which the effect of the covering liquid composition on the quality of the final product has been extensively studied [8,10,16,17,18,19], in the case of buffalo Mozzarella cheeses, such as MBC, this knowledge is lacking. For these reasons, because of the differences between buffalo and cow Mozzarella cheeses, e.g., the different fat-to-protein ratio, this study aimed to understand the relation between the composition of the covering liquid (i.e., % of NaCl, type of organic acid used) and the variation of some MBC quality properties (such as pH, electrical conductivity, color, expressible serum, texture, meltability, rheological properties) during a 30-day refrigerated storage period.

## 2. Materials and Methods

### 2.1. Experimental Trials

Eight batches of Mozzarella di Bufala Campana PDO (MBC) manufactured from March to September 2022 were used for this experiment; each batch of cheese was designated the technological replicate of the experimental design. The batches of cheeses (each composed of 192 cheese units) were collected from different Italian dairies affiliated to the PDO Consortium, to implement the expected variability related to the cheesemaking technology in the experimental design.

After cheesemaking, each batch of cheese was stored in six different covering liquids. The composition of the covering liquids varied in relation to the NaCl percentage (1, 2% *w*/*w*) and the type of organic acid used (citric acid, lactic acid, 1:1 lactic:citric acid, by modulating the acid addition to reach a titrable acidity of 6.5 °SH/50 mL). The samples were then stored at refrigeration temperature (4 ± 1 °C) and analyzed at four different storage times (2, 15, 22, 30 days after cheesemaking). The experimental trials (n = 192) were arranged as a full factorial design by considering the percentage of NaCl, the type of organic acid in the covering liquid, and the days of refrigerated storage as independent variables of the design.

### 2.2. Preparation of Covering Liquids

Food-grade citric acid monohydrate (Citrique Belge, Tienen, Belgium) and lactic acid (Chimlab, Padova, Italy), having a degree of purity of 90% and 80%, respectively, and food-grade NaCl was used for the preparation of the covering liquids. Firstly, bulk solutions of citric acid and lactic acid (1 N) were prepared by dissolving 389 g of citric acid monohydrate and 563 g of lactic acid, respectively, in tap water up to a final volume of 5 L. These bulk solutions were then diluted with tap water to a final concentration of ~2.28 g/L and ~0.98 g/L for citric and lactic acid, respectively. To do so, ~325 mL of citric acid 1 N solution and ~108 mL of lactic acid 1 N solution were diluted with tap water up to a volume of 10 L. The lactic:citric acid mix (1:1) was then prepared by mixing 3.2 L of both citric and lactic acid solutions, with an acidity of 6.5 °SH/50 mL. Finally, the 3 different solutions (citric acid, lactic acid, 1:1 lactic:citric acid) were divided into 2 aliquots with volume of 3.2 L each and added to NaCl up to a final NaCl concentration of 1 and 2% (*w*/*v*). The six obtained covering liquids, with an acidity of 6.5 °SH/50 mL (1%NaCl + citric acid; 2% NaCl + citric acid; 1% NaCl + lactic acid, 2% NaCl + lactic acid, 1% NaCl + lactic:citric acid 1:1, 2% NaCl + + lactic:citric acid 1:1), were then heat treated at 75 °C for 10 min and then cooled at ~4 °C, before being used to store the cheeses.

### 2.3. Cheesemaking and Storage

Batches of fresh MBC (weight ~125 g) were manufactured from heat-treated Mediterranean buffalo milk (thermized or pasteurized) according to the usual cheesemaking protocols performed by each of the eight different dairies considered in this study (Table 1), by following the criteria established by the Mozzarella di Bufala Campana PDO Regulation [20]. Briefly, cheeses were manufactured using fresh buffalo milk that was transformed within 60 h of milking; after the heat treatment, the milk was cooled to coagulation temperature (~37–39 °C). An aliquot of natural whey starter (3 ± 1% *v*/*v*) with a titrable acidity of 17 ± 1 °SH/50 mL, together with liquid calf rennet, were added to coagulate the milk. After milk coagulation and gel cutting, the cheese curd was left to acidify. Curd was then mechanically stretched when the pH reached a value of 4.89 ± 0.02. After stretching, the molten curd was molded by mechanical/automatic molding systems. Molded cheeses (with a temperature of 70 ± 5 °C) were then cooled by dipping in tap water (at ~15 °C) and then brine salted for up to 60 min. The cheeses, immediately after brining, were packed in rigid polypropylene containers together with 100 g of covering liquid (at ~10 °C). After packaging, the cheeses were immediately shipped at 6 ± 2 °C to the laboratories of the University of Parma, where they were stored at 4 ± 1 °C until they reached the predefined storage times. Before analyses, samples were taken out from the refrigerator and equilibrated in a climate chamber (ICH 256L, Memmert, Schwabach, Germany) at 25.0 ± 0.1 °C for 1 h [21].

### 2.4. Physical and Chemical Analyses

Changes in cheese weight (*WC*) during refrigerated storage were measured by a laboratory scale (BCE 5200, Orma, Milan, Italy) with an accuracy of ±0.1 g. Three cheeses for each sample were randomly selected and weighed at the beginning of the storage period (2 d after cheesemaking) and for all the following storage times (15, 22, 30 d after cheesemaking). The weight was measured by taking out each cheese and draining off the covering liquid; then, after weighing, the samples were re-immersed into their covering liquid and kept in refrigerated storage until the next sampling time. Weight variations were expressed as percentage changes of the original weight. For each sample, seven cheeses were monitored to track the changes in weight during the storage time.

Moisture Content (*MC*) of the cheeses was measured in triplicate by drying the sample (~3 g) until they reached constant weight by oven-drying, according to the AOAC method [22]. Each sample was measured at least in triplicate.

Colorimetric characteristics of the inner and outer part of the cheese were measured using a Minolta Colorimeter (CM 2600d, Minolta Co., Osaka, Japan) equipped with a D65 illuminant. The measurements were carried out at room temperature (25 °C). The CIELab color space was considered, and the parameters L* (lightness, black = 0, white = 100), a* (redness > 0, greenness < 0), b* (yellowness > 0, blue < 0) were determined by performing five replicated measurements for each sample. Each sample was measured at least in quintuplicate.

Expressible serum (*ES*) was measured in triplicate by centrifuging (12,500× *g* for 75 min) 30 g of sample in 50 mL laboratory tubes using a benchtop centrifuge (mod. 5810 R, Eppendorf, Hamburg, Germany) according to Guo et al. (1995) [23]. After centrifugation, the fat layer (supernatant) was removed by pipetting, the serum was transferred into another tube, and the quantity was weighed using an analytical scale (mod. AR 2140, Ohaus Corporation, Parsippany, NJ, USA). The analysis was performed at least in duplicate for each sample. *ES* was expressed as percentage of the weighted serum (*ES_app_*) related to the *MC* of the cheese, according to Equation (1):

*ES*% = (*ES_app_*/*MC*) × 100 (1)

pH and electrical conductivity of *ES* and covering liquid were, respectively, measured at 25 °C by means of Portamess pH-meter equipped with a Double Pore F electrode (Hamilton Company, Reno, NV, USA) and a conductometer mod. 913 (Knick Elektronische, Berlin, Germany) equipped with a TetraCon 325 probe (WTW Xylem Analytics, Weilheim, Germany) with a cell constant (K) of 0.475 cm^−1^. Both the analyses were performed in duplicate.

Optical density (*OD*) of the covering liquid was measured using a portable turbidimeter (Aqualytic mod. AL 200-PC, Lovibond, Amesbury, UK); to do so, 30 mL of covering liquid was diluted with 300 mL of distilled water (1:10 dilution). OD was expressed in NTU (Nephelometric Turbidity Unit). The measurements were performed in triplicate.

### 2.5. Texture Profile Analysis

Cheese texture was measured at room temperature using a TA.XT2plus texture analyzer (Stable Micro Systems, Godalming, UK). The analysis was performed on small cubes (1.5 × 1.5 × 1.5 cm) that had previously been cut with a knife. Texture Profile Analysis (TPA) was performed using a stainless-steel cylindrical probe with a diameter of 30 mm. Samples were compressed to 60% strain by applying a crosshead speed of 1.5 mm/s. Textural parameters considered were hardness (N), cohesiveness, springiness, and gumminess (N). Each sample was measured at least in quintuplicate.

### 2.6. Rheological Analyses

The rheological properties of the cheeses were assessed using a MCR 102 rheometer (Anton Paar, Gratz, Austria) equipped with 25 mm crosshatched parallel plates and a Peltier cell system (P-PTD 200, Anton Paar) connected to a thermostatic bath (Corio CD-200F, Julabo GmbH, Seelbach, Germany).

Disk-shape samples with a thickness of 6 mm and a diameter of 25 mm were gently cut from the central part of the cheese using a slicer and a borer. The disks were positioned between the parallel plates of the instrument at a fixed gap of 5 mm and covered with a solvent trap to prevent moisture loss during analysis. Before starting with the measurements, the samples were equilibrated at 25 °C for 5 min. Frequency sweep experiments were conducted at 25 °C in the angular frequency range between 1 and 100 rad s^−1^ by imposing a constant strain (0.05%) that fell within the linear viscoelastic range (LVR) of the samples (previously determined by strain sweep tests). The frequency dependence of storage modulus (G′) and loss modulus (G″) was modeled by power law equations [24]:

G′ = *k*′(*f*)*^n^*^′^ (2)

G″ = *k*″(*f*)*^n^*^″^ (3)

where *k*′ and *k*″ represent the magnitude of storage (G′) and loss (G″) moduli at a frequency of 1 rad s^−1^, while *n*′ and *n*″ indicate the frequency-dependence of rheological moduli. The ratio between the loss and the storage moduli (G″/G′, defined as tanδ) was also calculated within the frequency range.

Afterwards, temperature sweep tests were performed by applying a 0.5 °C min^−1^ temperature ramp, from 25 °C to 80 °C, at a constant angular frequency of 10 rad s^−1^ and strain of 0.05%. A total number of 55 acquisition points was acquired during frequency sweep experiments. From this experiment, the crossover temperature (*Tc*) of moduli (G′ = G″) was determined, which is indicative of the transition from the unmelted to the melted state of the cheese during heating [25]. Both rheological analyses were performed at least in duplicate.

### 2.7. Statistical Analysis

For all the analyzed parameters, the main effect of refrigerated storage time (2, 15, 22, 30 d), percentage of NaCl (1, 2%), the type of acidulant (lactic acid, citric acid, 1:1 mix of citric and lactic acid), and the significance of their interactions were evaluated considering a *p*-value ≤ 0.05 by mixed ANOVA models using PROC MIXED from SAS v.3.81 (SAS Institute Inc., Cary, NC, USA). The dairy (n = 8) was considered as the random factor of the models. Post hoc tests were performed with an LSMEANS statement by Fischer’s LSD test (α = 0.05) when significant main effects and interactions were found. Pearson’s correlation coefficients (r) were also calculated to find relations among evaluated variables using SPSS v.29 (IBM, Armonk, NY, USA).

## 3. Results and Discussion

### 3.1. Physico-Chemical Properties

The results of the mixed ANOVA models of the measured physico-chemical properties are reported in Table 2. As it is possible to observe, all the measured parameters, with the only exception of *EC_CL_*, L*_int_ and a*_int_, showed a significant main effect (*p* < 0.05) related to the storage time; the amount of NaCl in the covering liquid showed significant main effects (*p* < 0.05) for different measured parameters (*WC*, *MC*, *ES*, *EC_CL_*, *EC_ES_*, *pH_ES_*, and a*_int_), while the type of organic acid added to the covering liquid showed significant main effects (*p* < 0.05) for *pH_CL_*, *pH_ES_*, *OD_CL_*, a*_int_, and b*_int_.

**Figure 1:**
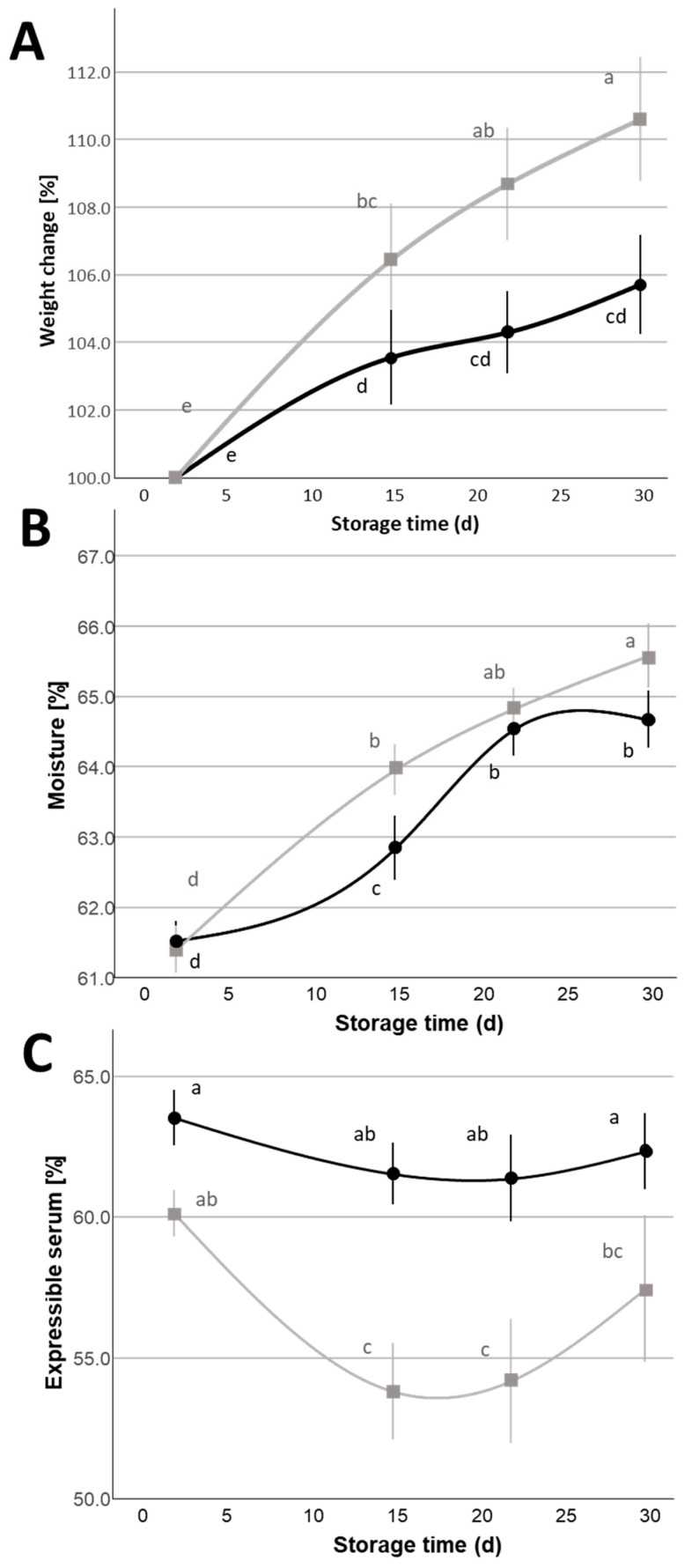
Weight change (**A**), moisture content (**B**), and expressible serum (**C**) variation (Mean ± Standard Error, n = 24) of Mozzarella di Bufala Campana PDO cheeses with covering liquids with different NaCl contents (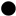 = 1%, 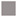 = 2%) during the 30 d refrigerated storage. Different letters indicate significant within-group differences (*p* < 0.05) based on LSD post hoc test.

As it is possible to observe from Figure 1A,B, both *WC* and *MC* showed an increase over the refrigerated storage of the cheeses that can be explained by water absorption phenomena from the covering liquid to the cheese [21,26,27], and also resulted in a moderate correlation between the two parameters (r = 0.324, *p* < 0.01 and n = 192) (Table S1).

The mass exchange between the cheese and covering liquid can be influenced by the characteristics of the cheese (i.e., porosity, composition, etc.), which can influence the diffusivity of solutes and water [28], but also by the composition of the covering liquid [8]. In particular, a higher NaCl content in the covering liquid (2%) caused a significantly (*p* < 0.05) higher moisture content and a higher weight increase (visible from 22 d of storage), resulting in a ~+5% weight at 30 d of storage compared to the samples containing 1% NaCl in the covering liquid. The utilization of a covering liquid containing 2% NaCl may result in cheeses not complying with the PDO moisture requirement (*MC* < 65%) if the duration of shelf life is prolonged to 30 d.

The fraction of unbound water contained in MBC cheese can be related to the sensory parameter of juiciness [26], and to the ability of the cheese, when cut, to exude whitish, fatty, and whey-like droplets with a lactic aroma, as stated by the PDO standard. This property can be estimated by measuring the expressible serum (*ES*). ES was significantly lower (*p* < 0.05) in the case of samples with covering liquid containing 2% NaCl (Figure 1C). These latter samples already showed significantly lower *ES* values at the beginning of refrigerated storage (2 d) until 22 d, while at the end of the storage period (30 d) they did not show a significant difference (*p* > 0.05). These results are in accordance with previous findings regarding buffalo-milk Mozzarella [29] and also with data related to both low- and high-moisture cow-milk Mozzarella cheeses [10,30,31,32], which highlighted a greater extent of casein swelling phenomena and an increase in the sub-micellar caseins’ fractions associated to salting in phenomena of cheeses having a higher amount of NaCl. In particular, the possible higher swelling of the protein matrix in the presence of a covering liquid with 2% NaCl means a higher amount of free water that becomes bound to the cheese domain (i.e., the casein network). The consequent decrease in free water (i.e., expressible serum) in the cheese domain probably favors the absorption phenomena of water from the covering liquid to the cheese and explains the measured increase in *MC*.

The *ES* variation over the evaluated storage time showed a decreasing trend that was significant (*p* < 0.05) between 2 and 15 d for the samples stored in covering liquid with 2% NaCl; this variation is also in accordance with previous reports from Gianferri et al. (2007) [33], who observed a decrease in the molecular mobility of serum water in Mozzarella di Bufala Campana PDO during a 14 d refrigerated storage period. In that case, the authors attributed the phenomenon to structural rearrangements of the protein domain that becomes more prone to interacting with the water molecules within the serum channels. These structural rearrangements were also described from a microstructural point of view by other authors [34], who observed an expansion of the casein matrix (i.e., the proteins became more hydrated, with an increase in their hydrodynamic volume) into the areas between fat globules, partially occupying the space previously filled by the interstitial serum between the closely packed fat globules.

Other factors may also be involved in this change, such as proteolytic phenomena that may increase the number of hydrophilic sites available for water coordination [35], but also the advancement of NaCl exchange between cheese and covering liquid. Experiments performed by Kruif et al. (2015) [36] on rennet-induced casein gels observed time-dependent swelling phenomena of these systems in low osmotic pressure solutions (<90 kPa) reaching an equilibrium in the order of days; this phenomenon was positively related to the casein volume fraction of the systems. Proteolytic phenomena in Mozzarella di Bufala Campana PDO cheeses have been mainly associated with the activity of plasmin, which is highly thermostable and can survive the milk heat treatments and the curd stretching operations [37]. Conversely, the occurrence of casein fragments that may be associated with the residual activity of rennet or psychrotrophic bacteria during cheese shelf life may be negligible if the milk and the curd are properly stored and managed, in accordance with the PDO standard [37].

The *ES* values of Mozzarella di Bufala Campana PDO at the beginning of the shelf life (~60–63%) were generally higher compared to Italian high-moisture Mozzarella cheeses made from cow milk and stored in covering liquid that showed values ~41–53% at the beginning of its shelf life, depending on the cheesemaking technology and cheese characteristics [24,38]; much lower values and highly variable values (~0–40%) were instead reported for Mozzarella cheeses not stored in covering liquid [23,31,32]. These differences in ES values of MBC compared to cow-milk Mozzarella may be mainly explained by diversities in composition; although the cheese moisture content of MBC may be similar to high-moisture Mozzarella, MBC has a fat-to-casein ratio of ~1.8, related to the higher fat-to-protein content of water buffalo milk compared to cow milk. Besides this effect, other factors that affect protein hydration such as protein-to-moisture ratio and calcium equilibria can be cited [39]. Furthermore, protein hydration properties may be also driven by differences in protein functionality (buffalo vs. cow caseins), cheesemaking technologies, and storage conditions (immersion in covering liquid vs. vacuum packaging).

Electrical conductivity is an untargeted, rapid, and non-expensive analysis that may be useful to keep track of the exchange of charged molecules between the cheese and the covering liquid. The electrical conductivity of *ES* (*EC_ES_*) showed a negative correlation with *ES* (r = −0.421, *p* < 0.01 and n = 192) (Table S1) and slightly increased over storage (Table 3), suggesting an increase in the electrical conductivity of the cheese, possibly associated with the migration of NaCl from the covering liquid to the cheese matrix, promoting NaCl-associated swelling and salting-in phenomena that may be responsible for a greater capability of the system to coordinate water.

Conversely, *EC_CL_* was not affected by the storage time (*p* > 0.05). Moreover, other than NaCl, the migration of different components present in the cheese can be responsible for the changes in electrical conductivity, such as organic acids, other minerals, and peptides [40]. Organic acids, as well as peptides containing basic or acidic aminoacidic residues (e.g., lysine, glutamic acid) are able to ionize depending on the pH of the medium and they can influence the electrical conductivity of the system, since this property is related to the presence and to the capability of ions to move within an electric field. Accordingly, the migration of NaCl from the covering liquid to the cheese may be also counterbalanced by the demineralization of caseins, as well as by proteolytic phenomena. Still, both *EC_ES_* and *EC_CL_* were obviously highly influenced (*p* < 0.05) by the different NaCl percentage added to the covering liquid, with 2% NaCl samples exhibiting much higher electrical conductivity values.

**Figure 2:**
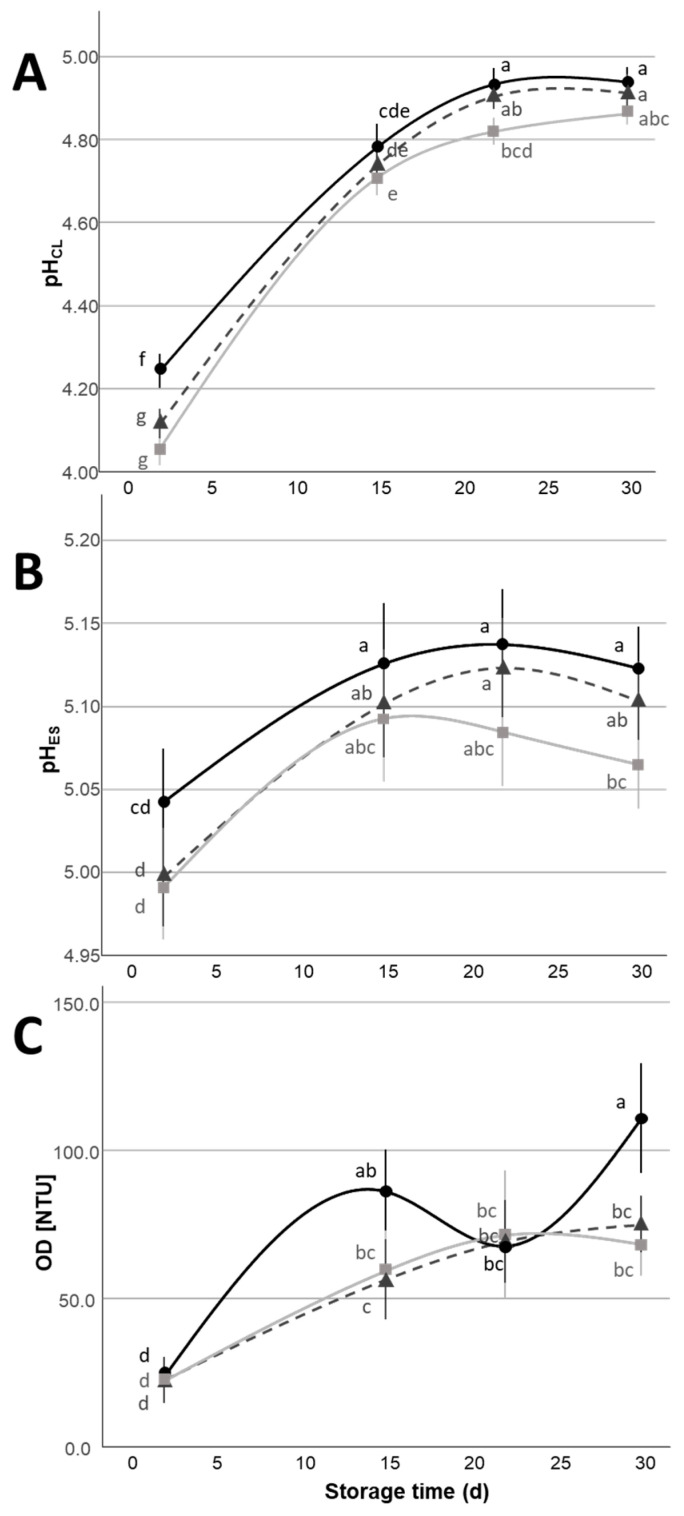
pH of covering liquid (*pH_CL_*) (**A**), of expressible serum (*pH_ES_*) (**B**), and optical density of covering liquid (**C**) variation (Mean ± Standard Error, n = 16) of Mozzarella di Bufala Campana PDO cheeses with covering liquids with different organic acids (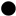 = citric acid, 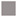 = lactic acid, 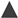 = 1:1 citric:lactic acid mix) during the 30 d refrigerated storage. Different letters indicate significant within-group differences (*p* < 0.05) based on LSD post hoc test.

The values of pH in both CL and ES (Figure 2A,B) showed a significant increase (*p* < 0.05) during the refrigerated storage, which was mostly evident during the first 15 d of storage; in particular, the pH varied from ~4.1 and ~5.0 for the initial storage time, to ~4.9 and ~5.1 for the final one, for CL and ES, respectively. Previous reports about high-moisture Mozzarella made with cow milk generally highlighted opposite variations of *pH_CL_* and *pH_ES_* caused by mass exchanges of organic acids [8,17] or a drop of pH during the refrigerated storage attributable to post-acidification phenomena caused by Lactic Acid Bacteria (LAB) growth [10,18,19,38,41]. In the present work, possible post-acidification phenomena driven by LAB were not appreciable by means of pH measurements, since the pH did not show a decrease during the whole storage time. Other authors [42,43] who evaluated the variation of pH in water buffalo Mozzarella observed an increase in the pH during the refrigerated storage. The increase in the pH of both CL and ES can then be associated with a modification of the buffering capacity of these systems that may be due to the gradual increase in para-casein hydration and the increased availability of various protein residues [44], and to casein solubilization phenomena due to proteolysis and a modification in mineral equilibria [32,45]. Also, a significant main effect related to the type of acid (*p* < 0.05) used for the covering liquid was observed for *pH_CL_* and *pH_ES_*, with citric acid causing higher pH values compared to the citric and lactic acid mixture, and to lactic acid, respectively. These differences can be explained by the different buffering capacities and the different numbers of acidic groups of lactic and citric acid, particularly if their addition is fixed in terms of final acidity in the covering liquid (6.5 °SH/50 mL). The lactic acid is characterized by one acid group with a pKa ~3.9, while the citric acid is characterized by three acid groups with pKa_1_ ~3.0, pKa_2_ ~4.1, and pKa_3_ ~4.8 in milk [46].

Thus, to reach the same titrable acidity level in the covering liquid, the normal concentration of citric acid is ~1/3 of lactic acid and the pKa_2_ and pKa_3_ are higher than the pKa of lactic acid, indicating a lower degree of dissociation in the pH values of the covering liquid (~4–5) and resulting in lower pH values in the case of the utilization of lactic acid compared to the mixture and the use of citric acid only. Also, in the case of *pH_ES_*, a significant main effect of the NaCl % in the covering liquid was present (*p* < 0.05) that may be attributed to a modification in the buffering capacity due to a different degree of calcium phosphate solubilization [47]; the *pH_ES_* difference caused by the NaCl % was low (*pH_ES_*~5.13 for samples with 1% NaCl vs. *pH_ES_*~5.07 for samples with 2% NaCl).

Covering liquid *OD* showed significant main effects related to both storage time and type of acid (*p* < 0.05); the *OD* increased during shelf life because of a gradual loss of solids (cheese particles, fat globules, proteins, minerals) from the cheese to the covering liquid, causing an increase in light scattering phenomena of the aqueous medium. The covering liquids containing citric acid had higher *OD* values than those made with lactic acid or the mix of citric and lactic acid (*p* < 0.05). Thybo et al. [48] demonstrated a higher ability of citric acid in chelating calcium compared to lactic acid. Thus, the stronger chelation of calcium by citric acid with formation of citrates may have influenced the structural characteristics of the cheese and improved the losses of solids over the shelf life [49], since a reduction in micellar calcium can be responsible for lowering the structural organization of the casein network [44] mainly at the cheese surface, thus favoring a possible loss of solids in the covering liquid.

As the MBC PDO standard establishes that the color of the cheese surface has to be white porcelain, this parameter may be important for assessing cheese quality and compliance with the regulation. The results given by the colorimetric analyses are reported in Table 4. The lightness (L*) of the cheese surface was higher than the interior because of the different physical appearance of the cheese skin, probably caused by the heating–cooling sequence during the stretching and hardening steps, leading to the typical smooth and brilliant surface. Also, the a* and b* values of the cheese surface were different from the interior ones, probably because of the effect of cheese hardening in water, which is potentially responsible for mass exchange phenomena. The same trend was also observed for high-moisture cow Mozzarella cheese [24].

As previously noted, the colorimetric coordinates were significantly influenced by the storage time (*p* < 0.05), except for L*_int_ and a*_int_ (Table 2). During the refrigerated storage, the surface of the cheese changed its colorimetric characteristics, highlighting a slight decrease in lightness (−L*_ext_) and a slight increase in greenish (−a*_ext_) and yellowish color (+b*_ext_). Conversely, in the inner part of the cheeses, the b* coordinate showed a decrease. Despite these significant differences, it should be mentioned that the total color difference (Δ*E*) calculated both in the inner and outer parts of the cheeses within the first storage time (2 d) and the subsequent storage points was lower than two, indicating color differences that could not be easily observed by the human eye. These variations could be due to compositional and structural modifications (e.g., moisture variation, protein swelling) that may cause a variation in the light scattering phenomena [24,50]. In support of these hypotheses, significant correlation coefficients were also found between the colorimetric coordinates and MC of the cheeses, such as for L*_ext_ (r = −0.320, *p* < 0.01) and b*_ext_ (r = 0.251, *p* < 0.01), but also with textural and rheological parameters (Table S1). Also, a*_int_ and b*_int_ were significantly influenced by the type of organic acid added to the covering liquid, with citric acid determining slightly lower values for both parameters (−2.50 ± 0.03 and 12.58 ± 0.12, respectively) compared to lactic acid (−2.43 ± 0.03 and 12.08 ± 0.12, respectively) and to the mixture of citric and lactic acid (−2.49 ± 0.03 and 12.44 ± 0.12, respectively). Also, the NaCl % in the covering liquid impacted on the a*_int_, with 2% NaCl promoting slightly but significantly (*p* < 0.05) lower a*_int_ (−2.52 ± 0.03) compared to 1% NaCl (−2.43 ± 0.03).

### 3.2. Textural and Rheological Properties

Knowledge of the textural and rheological characteristics of MBC PDO cheese is at present very poor and this report is one of the first describing these properties.

**Figure 3:**
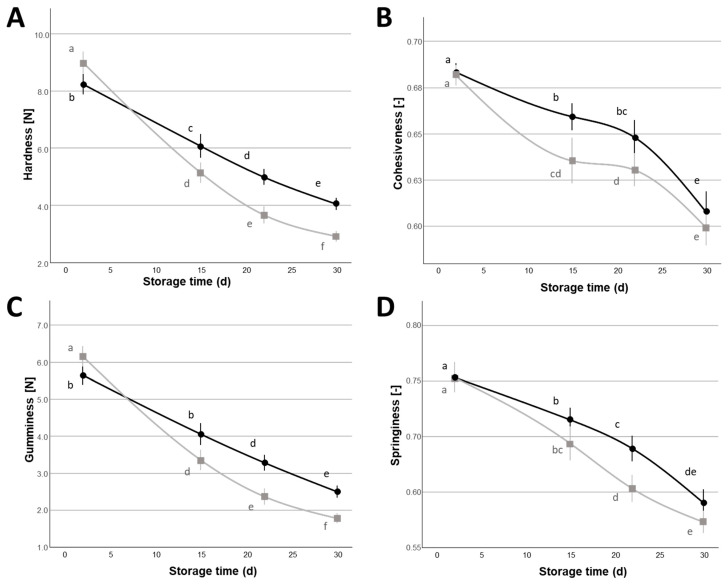
Hardness (**A**), cohesiveness (**B**), gumminess (**C**), and springiness (**D**) variation (Mean ± Standard Error, n = 24) of Mozzarella di Bufala Campana PDO cheeses with covering liquids with different NaCl contents (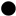 = 1%, 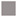 = 2%) during the 30 d refrigerated storage. Different letters indicate significant within-group differences (*p* < 0.05) based on LSD post hoc test.

All the evaluated textural parameters (hardness, cohesiveness, gumminess, springiness) showed significant main effects (*p* < 0.05) for both storage time and the percentage of NaCl used in the covering liquid (Table 5). Also, the power law parameters derived from frequency sweep measurements showed significant main effects (*p* < 0.05) for these two factors. Conversely, none of these textural and rheological parameters showed a significant effect of the type of organic acid used in the covering liquid (*p* > 0.05). The variation of textural properties (Figure 3) showed a decrease during the refrigerated storage period, particularly noticeable for hardness and gumminess, which decreased for more than 50% of the initial values, from the beginning to the end of the storage period; this phenomenon was also consistent with previous reports made on cow milk Mozzarella cheese [39,51].

**Figure 4:**
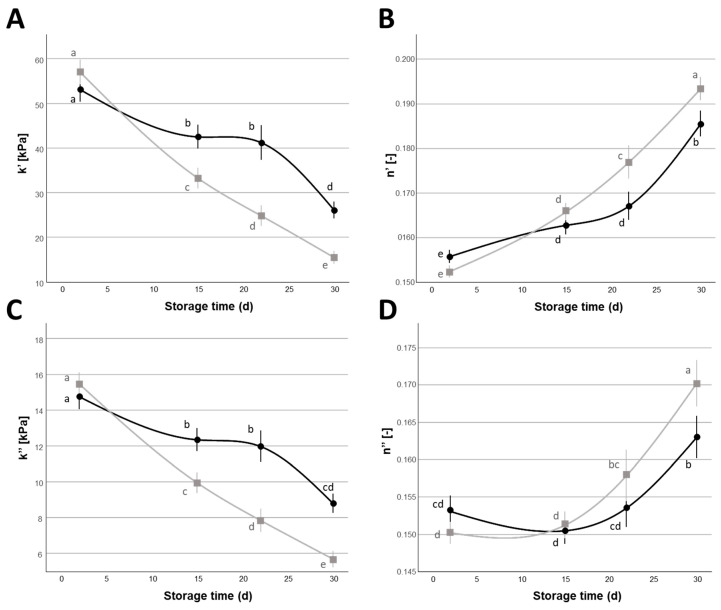
Variation of power law rheological parameters *k’* (**A**), *n’* (**B**), *k’’* (**C**), and *n’’* (**D**) (Mean ± Standard Error, n = 24) of Mozzarella di Bufala Campana PDO cheeses with covering liquids with different NaCl contents (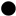 = 1%, 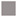 = 2%) during the 30 d refrigerated storage. Different letters indicate significant within-group differences (*p* < 0.05) based on LSD post hoc test.

Also, the rheological parameters *k*′ and *k*″, which indicate the quantity of the rheological moduli G′ and G″ at 1 rad s^−1^, showed a similar decrease during the refrigerated storage (Figure 4A,C). The rheological parameters *n*′ and *n*″, which indicate the frequency dependence of the moduli and are related to the type and strength of interactions that form the casein network [24], showed an increase during refrigerated storage (Figure 4B,D), which is indicative of a lowering of the internal bonds of the network. These rheological modifications were also coherent with previous reports made on Italian high-moisture Mozzarella cheese [38], which observed a decrease in *k*′ and *k*″ and an increase in *n*′ and *n*″ values over a 10 d refrigerated storage period. These textural and rheological changes can be directly influenced by the increase in moisture of the cheeses and to casein swelling phenomena during refrigerated storage. Moderate correlation coefficients between *MC*, hardness, and gumminess (r = −0.417 and −0.399, respectively, *p* < 0.01) or *MC*, *k*′, and *k*″ (r = −0.414 and −0.390, respectively, *p* < 0.01) support the relationship between moisture increase and partial destabilization of the cheese structure (Table S1). As is well known, water exerts a plasticizing effect on the physical domain formed by the casein network, thus decreasing the gel strength [25,52]. Also, proteolysis, which may be primarily caused by the activity of plasmin, and to a lower extent by rennet residual activity or by the activity of microbial exogenous proteases, may contribute to this decrease [14,22,35]. Finally, the mineral equilibria of Ca and P between the colloidal and soluble phase, which are influenced by the presence of NaCl and the pH, can influence the rheological properties of the cheese, since a reduction in colloidal calcium phosphate is often associated with a reduction in stiffness and a loss of structural integrity of the casein network [39,44].

As stated previously, the percentage of NaCl in the covering liquid significantly (*p* < 0.05) impacted on the textural and rheological properties, with samples stored in 2% NaCl showing lower textural values and rheological moduli compared to samples stored in 1% NaCl (Figure 3 and Figure 4). In the case of hardness and gumminess (Figure 3A,C), the higher percentage of NaCl caused a higher rate of variation during the storage time, especially in the first 15 d of storage: ~−43% (from 1 d to 15 d of storage), compared to ~−26% of samples stored in 1% NaCl for hardness, ~−45%, compared to ~−28% of samples stored in 1% NaCl for gumminess. This different behavior also explains the significant NaCl × storage time interaction observed in the case of the hardness and gumminess mixed ANOVA models (Table 5).

Similarly, the rheological parameters *k’*, *n’*, and *k’’* also showed a significant NaCl × storage time interaction, related to the different variation of these properties during refrigerated storage, as influenced by the different NaCl content of the covering liquid: samples stored in 1% NaCl showed a lower variation of these parameters, especially in the first 22 d of storage, compared to samples stored in 2% NaCl. For example, the reduction in *k’* values was ~42% for 2% NaCl samples compared to ~20% for 1% NaCl samples. According to the similar differences arising from the textural and rheological analysis, texture and rheological parameters were significantly correlated to each other (Table S1). The diverse impacts of the different percentages of NaCl on the textural and rheological properties may again reflect the modifications that involve the protein fraction. As mentioned in the previous paragraph (3.1), protein swelling phenomena and casein solubilization due to a change in mineral equilibria were possibly favored by the higher percentage of NaCl in the covering liquid, leading to higher moisture-to-protein ratio, a greater plasticizing effect of water on the casein fibers, and a lower degree of structural order of the network. These results can be interpreted in a similar manner to previous reports from Bähler et al. [53], who observed a decrease in the hardness of Mozzarella cheeses as a consequence of higher NaCl content in hot brining operations, due to an increase in the moisture-to-protein ratio of the system; other reports also showed a significant reduction in the cheese’s textural and rheological characteristics when the NaCl content of Mozzarella cheese was higher [39,54]. Swelling has an impact on the casein micelles gels’ voluminosity, with higher values resulting in a softer texture compared to casein gels with a lower water content and reduced diameter [53]. Moreover, proteolytic phenomena may also be influenced by the NaCl concentration [55].

**Figure 5:**
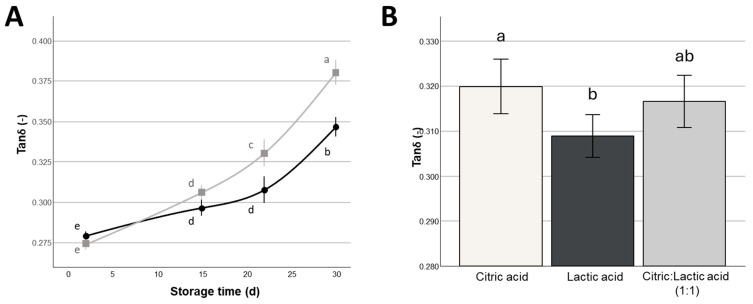
Variation of tanδ as a function of storage time and content of NaCl in the covering liquid (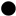 = 1%, 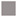 = 2%) (**A**) (Mean ± Standard Error, n = 24), and as a function of the type of organic acid added to the covering liquid (**B**) (Mean ± Standard Error, n = 64) of Mozzarella di Bufala Campana PDO cheeses. Different letters indicate significant within-group differences (*p* < 0.05) based on LSD post hoc test.

Also, *tanδ* values (Figure 5A) highlighted significant main effects of both storage time and the percentage of NaCl and significant NaCl × storage time interaction (*p* < 0.05), as the viscous-to-elastic behavior showed a greater increase over the refrigerated storage period of Mozzarella stored in 2% NaCl compared to 1% NaCl, indicative of a greater decrease in the solid-like behavior of the former samples. *Tanδ* also showed a significant main effect (*p* < 0.05) of the type of organic acid present in the covering liquid (Figure 5B), with citric acid promoting higher values compared to lactic acid. This difference may be related to the differences in pH discussed in paragraph 3.1, which may be related to differences in mineral equilibria and/or in proteolytic phenomena. Accordingly, *tanδ* showed also a significant correlation with *pH_CL_* (r = 0.483, *p* < 0.01).

**Figure 6:**
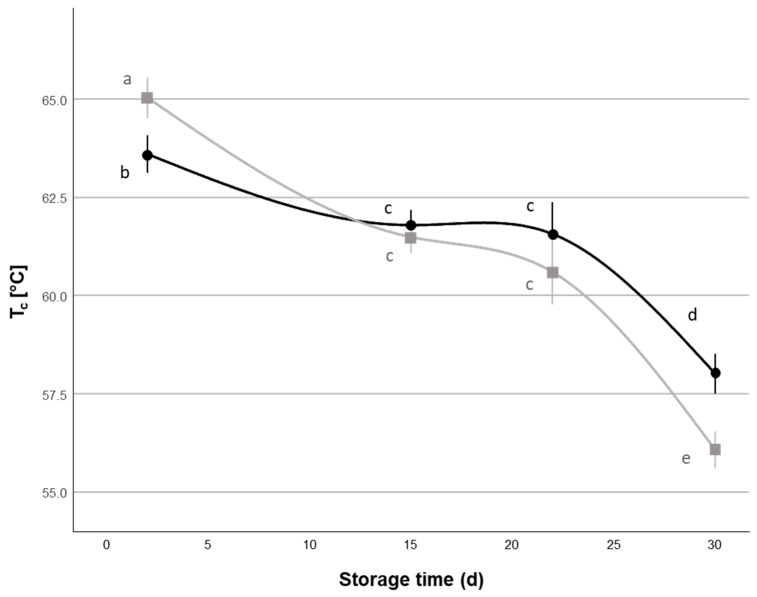
Variation of meltability evaluated by crossover temperature (*T_c_*) obtained from temperature sweep experiments (Mean ± Standard Error, n = 24) of Mozzarella di Bufala Campana PDO cheeses with covering liquids with different NaCl contents (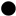 = 1%, 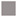 = 2%) during the 30 d refrigerated storage. Different letters indicate significant within-group differences (*p* < 0.05) based on LSD post hoc test.

Concerning the cheese melting behavior expressed as crossover temperature (*T_c_*), statistical analysis (Table 5) showed a significant main effect for storage time; also, it was possible to observe a significant NaCl × storage time interaction (*p* < 0.05), despite the NaCl main factor not showing a significant main effect (*p* > 0.05). As is possible to observe from the data (Figure 6), *T_c_* showed a decrease over the storage time. The increased meltability of Mozzarella cheese during storage has already been reported before [25,39,56]; this phenomenon has been mainly attributed to proteolytic phenomena that reduce the amount of intact casein and the temperature range over the solid-like behavior of the cheese dominates, but some changes in calcium-to-casein equilibria and the increase in casein hydration phenomena may also be determining factors [25,32,39,56,57]. When a higher percentage of NaCl was added to the covering liquid (2%), *T_c_* showed a stronger decrease (from ~65.0 °C to ~56.1 °C) over the evaluated storage period compared to 1% NaCl samples (from ~63.6 °C to 58.0 °C).

In particular, in this case, the effects that the percentage of NaCl in the covering liquid had on cheese meltability may be directly linked to the changes in protein hydration and voluminosity, which may decrease the distance between protein fibers, enhance protein–protein interactions, and thus decrease cheese meltability [25,31,56]. Accordingly, *T_c_* values were significantly correlated with the *MC* of the cheeses (r = −0.372, *p* < 0.01) (Table S1).

## 4. Conclusions

The composition of the covering liquid used to preserve the quality of Mozzarella di Bufala Campana PDO cheese during its shelf life showed a significant impact on several physical and chemical parameters evaluated during the 30 d refrigerated storage period. The behavior of these changes was not linear within the considered storage time. In particular, the NaCl content had the most major impact, with the highest NaCl percentage (2%) promoting an enhancement in the moisture adsorption and its related casein swelling phenomena, leading to a marked change in the textural and rheological parameters. Furthermore, moisture adsorption is a critical parameter because a moisture content of 65% is the legal maximum value accepted by the Mozzarella di Bufala Campana PDO cheese standard; for this reason, a deeper understanding of the kinetics of moisture adsorption related to covering liquid composition will play a pivotal role in allowing cheesemakers to find an equilibrium between the need to have the highest real cheese yield at the end of cheesemaking and the shelf life duration. The different types of acidulant used by dairies to regulate the acidity of the covering liquid had a lower impact on the evaluated physical and chemical properties of the cheese. The results of this study may be useful for cheesemakers to select a covering liquid composition to optimize the quality characteristics of the cheese and its real cheese yield within the expected shelf-life duration, also considering the requirements of the PDO standard.

## Figures and Tables

**Table 1:** Descriptive statistics (mean, standard deviation, coefficient of variation, minimum and maximum values) of the main cheesemaking operations performed by the different Mozzarella di Bufala Campana PDO dairies (n = 8) considered in the study.

Operation	Mean	SD	CV%	Min	Max
Milk heat treatment temperature (°C)	69.3	4.6	6.6	60.0	73.0
Milk heat treatment time (s)	20.0	8.7	43.3	15.0	30.0
Natural Whey Starter (L/100 L milk)	3.0	0.7	22.1	2.0	4.2
Natural Whey Starter acidity (°SH/50 mL)	16.8	0.9	5.1	16.0	18.5
Rennet (mL/100 L milk)	7.0	0.9	13.2	6.0	8.3
Rennet strength (IMCU)	220.0	16.4	7.5	205.0	235.0
Time from rennet addition to coagulum cutting (min)	51.3	26.6	51.8	20.0	90.0
Curd acidification time (from starter addition to stretching) (min)	278.1	32.6	11.7	240.0	320.0
Curd pH	4.9	0.0	0.6	4.9	5.0
Stretching fluid temperature (°C)	94.5	1.4	1.5	93.0	97.0
Stretching time (min)	7.5	6.0	80.5	3.0	20.0
Cheese temperature after molding (°C)	70.3	4.6	6.5	62.0	77.0
Hardening water temperature (°C)	14.9	3.8	25.3	10.0	20.0
Hardening time (min)	39.3	27.3	69.5	15.0	90.0
Brine temperature (°C)	11.6	3.6	30.8	5.0	16.0
Brining time (min)	45.0	16.9	37.6	20.0	60.0
Cheese temperature after brining (°C)	18.1	2.4	13.0	15.0	22.0

**Table 2:** *p*-values obtained from mixed ANOVA models of the evaluated physical and chemical parameters of Mozzarella di Bufala Campana PDO cheeses for each considered factor: storage time, NaCl, organic acid, and the interactions among them.

Factor	*WC*	*MC*	*ES*	*EC_CL_*	*EC_ES_*	*pH_CL_*	*pH_ES_*	*OD_CL_*	L*_int_	a*_int_	b*_int_	L*_ext_	a*_ext_	b*_ext_
Storage time	**<0.001**	**<0.001**	**0.029**	0.247	**<0.001**	**<0.001**	**<0.001**	**<0.001**	0.452	0.512	**<0.001**	**<0.001**	**<0.001**	**<0.001**
NaCl	**0.009**	**0.038**	**<0.001**	**<0.001**	**<0.001**	0.110	**0.029**	0.115	0.316	**0.004**	0.561	0.970	0.826	0.145
Organic Acid	0.487	0.527	0.962	0.770	0.751	**0.001**	**0.009**	**0.040**	0.268	**0.032**	**0.023**	0.924	0.266	0.799
Storage time × NaCl	**0.015**	0.186	0.428	0.478	0.203	0.951	0.540	0.083	0.803	0.875	0.253	0.941	0.864	0.973
Storage time × Organic Acid	0.617	0.082	0.444	0.995	0.429	0.465	0.935	0.171	0.324	0.146	**0.012**	0.128	0.754	0.897
NaCl × Organic Acid	0.092	0.259	0.803	0.708	0.954	0.994	0.164	0.238	0.523	0.998	0.985	0.805	0.168	**0.036**
NaCl × Organic Acid × Storage time	0.999	0.560	0.933	0.749	0.216	0.947	0.754	0.230	0.321	0.847	0.954	0.692	0.537	0.433

Abbreviations: relative weight change of the cheese (*WC*), moisture content (*MC*), expressible serum (*ES*), electrical conductivity and pH of covering liquid (*EC_CL_*, *pH_CL_*) and expressible serum (*EC_ES_*, *pH_ES_*), optical density of covering liquid (*OD_CL_*), external and internal colorimetric coordinates (L*_int_, a*_int_, b*_int_ and L*_ext_, a*_ext_, b*_ext_). *p*-values reported in bold highlight significant main effects or interactions (*p* < 0.05).

**Table 3:** Variation of electrical conductivity of expressible serum (*EC_ES_*) and covering liquid (*EC_CL_*) for Mozzarella di Bufala Campana PDO cheese samples as a function of NaCl content (1, 2%) and refrigerated storage (2, 15, 22, 30 d).

% NaCl	Storage Time (d)	EC_ES_ [mS]	EC_CL_ [mS]
1%	2	14.8 ± 0.3 ^e^	15.4 ± 0.3 ^b^
	15	15.6 ± 0.3 ^de^	15.5 ± 0.5 ^b^
	22	15.8 ± 0.5 ^de^	15.2 ± 0.3 ^b^
	30	16.9 ± 0.3 ^d^	16.1 ± 0.3 ^b^
2%	2	20.6 ± 0.5 ^c^	24.8 ± 0.6 ^a^
	15	23.1 ± 0.6 ^ab^	24.5 ± 0.8 ^a^
	22	23.0 ± 0.8 ^b^	23.8 ± 0.5 ^a^
	30	24.3 ± 0.5 ^a^	24.4 ± 0.3 ^a^

^a–e^ Samples’ subgroups with mean values with different superscript letters are significantly different (*p* < 0.05) based on LSD post hoc test. Values are expressed as Mean ± Standard error (n = 24).

**Table 4:** Variation of colorimetric coordinates measured on the inner part (L*_int_, a*_int_, b*_int_) and on the surface (L*_ext_, a*_ext_, b*_ext_) of Mozzarella di Bufala Campana PDO cheese samples as a function of refrigerated storage (2, 15, 22, 30 d).

Storage Time (d)	L*_int_	a*_int_	b*_int_	L*_ext_	a*_ext_	b*_ext_
2	92.92 ± 0.04 ^a^	−2.46 ± 0.01 ^a^	12.93 ± 0.05 ^a^	95.53 ± 0.02 ^a^	−1.64 ± 0.01 ^a^	6.79 ± 0.04 ^c^
15	93.12 ± 0.04 ^a^	−2.48 ± 0.01 ^a^	12.34 ± 0.05 ^b^	95.33 ± 0.02 ^b^	−1.71 ± 0.01 ^b^	7.18 ± 0.04 ^b^
22	93.15 ± 0.03 ^a^	−2.50 ± 0.01 ^a^	12.32 ± 0.04 ^b^	95.16 ± 0.02 ^c^	−1.78 ± 0.01 ^c^	7.73 ± 0.04 ^a^
30	93.09 ± 0.04 ^a^	−2.46 ± 0.01 ^a^	11.87 ± 0.05 ^c^	94.97 ± 0.02 ^d^	−1.78 ± 0.01 ^c^	7.81 ± 0.04 ^a^

^a–d^ Samples’ subgroups with mean values with different superscript letters are significantly different (*p* < 0.05) based on LSD post hoc test. Values are expressed as Mean ± Standard error (n = 48).

**Table 5:** *p*-values obtained from mixed ANOVA models of the evaluated textural and rheological parameters of Mozzarella di Bufala Campana PDO cheeses for each considered factor: storage time, NaCl, organic acid, and the interactions among them.

Factor	Hardness	Gumminess	Cohesiveness	Springiness	*T_c_*	*k*′	*n*′	*k*″	*n*″	*Tanδ_1rad/s_*
Storage time	**<0.001**	**<0.001**	**<0.001**	**<0.001**	**<0.001**	**<0.001**	**<0.001**	**<0.001**	**<0.001**	**<0.001**
NaCl	**0.004**	**0.004**	**0.016**	**0.016**	0.187	**0.001**	**0.019**	**0.001**	0.175	**0.002**
Organic Acid	0.434	0.394	0.797	0.353	0.101	0.889	0.774	0.987	0.381	**0.041**
Storage time × NaCl	**0.001**	**0.001**	0.257	0.222	**0.005**	**0.001**	**0.016**	**0.002**	0.145	**0.002**
Storage time × Organic Acid	0.533	0.519	0.512	0.104	0.999	0.260	0.094	0.342	0.418	0.136
NaCl × Organic Acid	0.938	0.898	0.296	0.286	0.198	0.577	0.154	0.318	**0.016**	0.615
NaCl × Organic Acid × Storage time	0.963	0.946	0.407	0.106	0.999	0.680	0.887	0.668	0.839	0.469

Abbreviations: crossover temperature (*T_c_*), loss factor measured at 1 rad/s (*Tanδ_1rad/s_*), power law regression parameters from storage modulus (*k*′, *n*′), loss modulus (*k*″, *n*″). *p*-values reported in bold highlight significant main effects or interactions (*p* < 0.05).

## Data Availability

The raw data supporting the conclusions of this article will be made available by the authors on request.

## Supplementary Materials

The following supporting information can be downloaded at: https://www.mdpi.com/article/10.3390/foods14091506/s1, Table S1. Pearson correlation coefficients of all the measured parameters of Mozzarella di Bufala Campana PDO samples.
