# Supplementary material for: Structural and Physico-Chemical Changes of Mozzarella di Bufala Campana Cheese Influenced by Covering Liquid Composition
Source: Foods. 2025 Apr 25;14(9):1506. doi: 10.3390/foods14091506 (PMC12071588; doi:10.3390/foods14091506)
Supplement: Supplementary file 1 [file foods-14-01506-s001.zip › foods-3568921-supplementary.pdf]

Table S1. Pearson correlation coefficients of all the measured parameters of Mozzarella di Bufala Campana PDO samples.

Parameter	WC	MC	ES	OD_{CL}	EC_{CL}	EC_{ES}	pH_{CL}	pH_{ES}	L^*_{int}	a^*_{int}	b^*_{int}
**MC**	0.324**	--									
**ES**	0.236**	-0.030	--								
**OD_{CL}**	0.591**	0.249**	0.133	--							
**EC_{CL}**	0.113	0.036	-0.395**	-0.055	--						
**EC_{ES}**	0.107	0.152*	-0.421**	-0.075	0.754**	--					
**pH_{CL}**	0.456**	0.567**	-0.087	0.415**	-0.127	0.070	--				
**pH_{ES}**	0.574**	0.336**	0.257**	0.483**	-0.158*	-0.297**	0.492**	--			
**L^*_{int}**	-0.145*	-0.145*	-0.131	0.050	0.080	0.151*	-0.126	-0.232**	--		
**a^*_{int}**	-0.016	-0.280**	0.024	0.052	-0.110	-0.071	-0.133	-0.201**	0.467**	--	
**b^*_{int}**	0.154*	0.021	0.172*	-0.009	-0.060	-0.206**	-0.108	0.275**	-0.628**	-0.678**	--
**L^*_{ext}**	-0.218**	-0.320**	0.016	-0.306**	0.081	0.026	-0.427**	-0.331**	0.284**	0.073	-0.022
**a^*_{ext}**	-0.088	-0.371**	-0.017	-0.176*	0.041	-0.101	-0.311**	-0.138	0.064	0.252**	0.043
**b^*_{ext}**	0.208**	0.251**	-0.044	0.318**	-0.081	0.031	0.337**	0.152*	0.159*	0.085	-0.174*
**Hardness**	-0.252**	-0.417**	0.257**	-0.286**	-0.176*	-0.354**	-0.607**	-0.088	-0.289**	-0.036	0.433**
**Gumminess**	-0.223**	-0.399**	0.274**	-0.274**	-0.187**	-0.357**	-0.597**	-0.053	-0.298**	-0.045	0.433**
**Cohesiveness**	0.082	-0.191**	0.402**	-0.100	-0.246**	-0.358**	-0.359**	0.213**	-0.269**	-0.083	0.364**
**Springiness**	-0.212**	-0.294**	0.272**	-0.310**	-0.204**	-0.280**	-0.433**	-0.051	-0.318**	-0.107	0.373**
**T_c**	-0.473**	-0.372**	-0.015	-0.424**	-0.003	-0.123	-0.516**	-0.273**	-0.114	-0.067	0.247**
**k'**	-0.290**	-0.414**	0.180*	-0.169*	-0.192**	-0.360**	-0.489**	-0.059	-0.240**	-0.058	0.335**
**n'**	0.248**	0.466**	-0.151*	0.208**	0.105	0.255**	0.485**	0.121	0.178*	-0.042	-0.293**
**k''**	-0.240**	-0.390**	0.214**	-0.134	-0.221**	-0.388**	-0.454**	-0.001	-0.258**	-0.067	0.350**
**n''**	0.121	0.409**	-0.032	0.069	0.036	0.109	0.221**	0.067	0.085	-0.135	-0.121
**$Tan\delta_{1rad/s}$**	0.311**	0.452**	-0.150*	0.250**	0.159*	0.293**	0.483**	0.135	0.211**	0.022	-0.316**

**The correlation is significant at a p-value < 0.01 (two-tailed);
*The correlation is significant at a p-value < 0.05 (two-tailed).

Table S1. Continued.

Parameter	L^*_{ext}	a^*_{ext}	b^*_{ext}	Hardness	Gumminess	Cohesiveness	Springiness	T_c	k'	n'	k''	n''
**MC**												
**ES**												
**OD_{CL}**												
**EC_{CL}**												
**EC_{ES}**												
**pH_{CL}**												
**pH_{ES}**												
**L^*_{int}**												
**a^*_{int}**												
**b^*_{int}**												
**L^*_{ext}**	--											
**a^*_{ext}**	0.298**	--										
**b^*_{ext}**	-0.329**	-0.761**	--									
**Hardness**	0.148*	0.376**	-0.510**	--								
**Gumminess**	0.146*	0.364**	-0.509**	0.995**	--							
**Cohesiveness**	0.169*	0.231**	-0.377**	0.691**	0.745**	--						
**Springiness**	0.183*	0.257**	-0.463**	0.800**	0.812**	0.722**	--					
**T_c**	0.200**	0.276**	-0.433**	0.630**	0.619**	0.438**	0.614**	--				
**k'**	0.158*	0.248**	-0.371**	0.693**	0.690**	0.552**	0.584**	0.700**	--			
**n'**	-0.202**	-0.294**	0.392**	-0.683**	-0.679**	-0.552**	-0.619**	-0.782**	-0.849**	--		
**k''**	0.138	0.226**	-0.342**	0.665**	0.663**	0.555**	0.550**	0.647**	0.989**	-0.820**	--	
**n''**	-0.092	-0.252**	0.239**	-0.339**	-0.326**	-0.281**	-0.304**	-0.503**	-0.609**	0.798**	-0.616**	--
**$Tan\delta_{1rad/s}$**	-0.199**	-0.295**	0.428**	-0.714**	-0.710**	-0.566**	-0.682**	-0.837**	-0.837**	0.939**	-0.800**	0.701**

**The correlation is significant at a p-value < 0.01 (two-tailed);
*The correlation is significant at a p-value < 0.05 (two-tailed).
